# A Study on sEMG-Based Motor Variability and Functional Connectivity of the Upper Limb Depending on Weight Distributions in a Handle of a Cordless Stick-Type Vacuum Cleaner

**DOI:** 10.3390/s22134835

**Published:** 2022-06-26

**Authors:** Hayeon Yu, Eunchae Kang, Joonho Chang

**Affiliations:** Department of Industrial and Systems Engineering, Dongguk University, Seoul 04620, Korea; hayou1620@dgu.ac.kr (H.Y.); kech305@naver.com (E.K.)

**Keywords:** cordless stick-type vacuum cleaner, center of mass of a handle, EMG analysis, motor variability, functional connectivity

## Abstract

This study investigated the muscle activities, motor variability, and functional connectivity of the upper limb as a function of weight distributions in a handle of a cordless stick-type vacuum cleaner. Eighteen female college students with experience of vacuum cleaner-use participated in testing. Five handles with different centers of mass (CM) were prepared (centroid, top-rear, top-front, bottom-front, and bottom-rear), and electromyography for the muscles of the upper limb were measured during vacuuming. The results showed that the %MVC values of the Extensor Carpi Ulnaris (*p* = 0.0038) and Deltoid Middle (*p* = 0.0094) increased but that of the Biceps Brachii (*p* = 0.0001) decreased, as the CM moved from the top to bottom area of the handle. The motor variability of the Extensor Carpi Ulnaris (*p* = 0.0335) and Brachioradialis (*p* = 0.0394) significantly varied depending on the CM locations but failed to show significance in the post-hoc analyses. Lastly, the functional connectivity values of the muscle pairs such as the Extensor Carpi Ulnaris–Deltoid Middle (*p* = 0.0016), Extensor Carpi Ulnaris–Upper Trapezius (*p* = 0.0174), Brachioradialis–Biceps Brachii (*p* = 0.0356), and Biceps Brachii–Upper Trapezius (*p* = 0.0102) were significantly altered as a function of the CM locations. The lowest functional connectivity was found with the handle of which CM was at centroid.

## 1. Introduction

As cordless vacuum cleaners are becoming steadily prevalent in the market, their market share has grown rapidly worldwide. Reportocean [1] reported that the global cordless vacuum cleaner market was $5780 million (USD) in 2021 and that it was forecasted to grow to $12,736.98 million (USD) by 2030. Two types of cordless vacuum cleaners have been typically introduced in the market: (1) a stick-type where a main body including a motor, a dust bag, a battery, etc., is attached near the top of a canister (i.e., handle) and (2) an upright-type where a main body is installed from the middle to bottom of a canister. The popularity of the cordless stick-type vacuum cleaners in particular has been increasing, as the benefits of its compact design become well-known [2,3]. The cordless stick-type vacuum cleaner market is expected to gradually grow by as much as 10.39% worldwide from 2021 to 2026, which seems to be regarded as the highest growth rate in the global cordless vacuum cleaner market [4,5].

Although the compact design of a cordless stick-type vacuum cleaner has contributed to the improvement of portability, convenience, and the efficacy of space utilization in its use, such design trends have been inducing potential usability issues in terms of design and structure. In particular, the handle of a cordless stick-type vacuum cleaner becomes relatively heavier than that of other types of vacuum cleaners, in addition to the fact that it is likely to have asymmetric weight distribution inside [6]. The reason is that components including a motor, a battery, and a dust bag need to be installed all together in the handle in order to maintain design compactness. This heavy handle, which is even likely to have asymmetric weight distribution, could have adverse effects on vacuuming performance as well as user experience. Kang et al. [6] pointed out that while using a cordless stick-type vacuum cleaner, users tend to prefer a handle, of which the center of mass (CM) is near its centroid, to the handles with asymmetric weight distribution.

Use of a cordless stick-type vacuum cleaner is likely to expose users to the risks of musculoskeletal injuries than would conventional vacuum cleaners. Above all, since identical cleaning motions and tasks are basically shared during vacuuming with both of the cleaners, the chronic problems of vacuuming including overexertion, bodily reaction, and repetitive motions related to musculoskeletal disorders (MSDs) still remain [7], as well-known, repetitive and excessive body movements lead to muscle injuries and further progress to MSDs, especially in their prolonged use [8,9]. In addition, the nature of the handles of a cordless stick-type vacuum cleaner could aggravate those potential risks of developing MSDs, because the heavier the handle the more effort that is required; Choi and Shin [10] revealed that using a cordless vacuum cleaner requires greater muscle effort of the upper limb during vacuuming rather than the use of conventional vacuum cleaners. In sum, although a cordless stick-type vacuum cleaner has primarily been used for housekeeping with relatively less frequency and short time of use as compared to vacuum cleaners for occupational purposes [11,12], studies are still needed to shed light on this issue to identify the extent of the MSD risks and to devise appropriate countermeasures from a biomechanical point of view.

Unfortunately, studies examining the potential risks of musculoskeletal injuries while using a cordless stick-type vacuum cleaner have almost never been conducted. A few of studies on the weight or CM of the handle of a cordless stick-type vacuum cleaner are being introduced. Choi and Shin [10] investigated the effects of the CM locations of cordless vacuum cleaners and confirmed that higher physical loads to a user’s upper extremity muscles were required during vacuuming when using the cordless vacuum cleaner, of which CM was near the handle. Qin et al. [13] examined the effect of the CM positions (above, front, and bottom areas of the handle) of a cordless stick-type vacuum cleaner handle on the muscle activities of the upper limb and revealed that there was no significant difference among those CM locations during floor vacuuming. More recently, Kang et al. [6] studied the variation of the muscle activity and subjective discomfort of the upper limb depending on weight distributions inside the handle of a cordless stick-type vacuum cleaner, and advised that the closer the CM is to the hand that grasps the handle, the greater its design benefit could become. In short, these researchers focused on the effect of the handle weight of a cordless stick-type vacuum cleaner and its weight distribution on the muscle activities of the upper limb and tried to provide the optimal designs. In addition, since most of these studies have been conducted primarily in terms of the short-term use, unfortunately it can be concluded that there have been little opportunities to examine the potential risks of developing MSDs that could be caused in the prolonged use of a cordless stick-type vacuum cleaner.

The analyses of motor variability and functional connectivity could be appropriate tools to examine potential adverse effects that could be caused in the long-term use of a cordless stick-type vacuum cleaner, because they have proven their performance in assessing the MSD risks of tasks in terms of muscle activity—in particular, these analyses have the advantage that they are able to be conducted directly based on the electromyography (EMG) signals obtained from task execution. In general, the motor variability represents the natural variation in postures, movements, and muscle activity that are observed at different levels of behavioral outcomes over time [14,15] and typically quantified using statistical indices such as the standard deviation (SD), median absolute deviation, coefficient of variation (CV), and inter-quartile range of EMG signals [15,16,17,18]. Since it typically indicates the extent to which a muscle has opportunities for muscular rest (i.e., period of relaxation) during task execution [19,20,21], researchers addressed that the higher the motor variability of a task is, the less the muscle fatigue develops during the task execution, which could help reduce the risks of developing MSDs [22,23,24,25]. The motor variability analysis has been used often in EMG studies, and in helping assess potential MSD risks of their tasks. For instance, it has been practically applied to a variety of tasks including occupational meat cutting [26], repetitive reaching back and forth between two cylindrical touch-sensitive targets [27], repetitive pointing at shoulder height [28], typing with sitting and walking [29], computer tasking (e.g., reading, typing) with dual monitors [30], keyboard typing with cycling [31] etc., and showing its validity as an index to assess muscle fatigue and potential MSD risks.

Next, the functional connectivity describes synergistic synchronization (i.e., intermuscular coherence) between muscles during the execution of motor tasks and has been suggested to assess muscle fatigue and find out injury mechanisms [32] Normalized mutual information (NMI) is typically employed as a computational technique to quantify the functional connectivity between muscles [30]. The NMI is defined as the amount of coordination patterns between muscles and accounts for both linear and non-linear relationships between two EMG time series, based on joint probability that is a measure of two events happening at the same time [33,34,35]. Since it simply indicates how much shared activation (i.e., EMG signal commonality) exists between two muscles, most of relevant studies have warned that the higher the NMI value between two muscles is, the more the muscle fatigue develops, which may elevate the risks of developing MSDs on those muscles [34,36,37]. The NMI has only recently been applied to EMG studies [30], and thus a large number of studies have not been conducted yet. There have been a few representative studies. For example, the applications have been practically done to EMG studies including dynamic tracking tasks using compensatory and pursuit display [38], repetitive pointing at shoulder height [28], texting on a smartphone and computer typing [39], computer tasking (e.g., reading, typing) with dual monitors [30], keyboard typing with cycle pedaling [31], etc. In sum, the NMI analysis has been proving its performance as an index to quantify EMG signal commonality between muscles, as well as being primarily used to predict their muscle fatigue and potential risks of developing MSDs.

This study investigated the muscle activities (i.e., %maximum voluntary contraction = %MVC), motor variability (i.e., CV), and functional connectivity (i.e., NMI) for the muscles of the upper limb as a function of weight distributions in a handle of a cordless stick-type vacuum cleaner, and thereby discussed the potential risks of developing MSDs, especially in its prolonged use. Three research hypotheses were tested: (1) weight distributions in the handle significantly affect the %MVC values of the muscles in the upper limb; (2) weight distributions in the handle significantly affect the CV values of the muscles in the upper limb; and (3) weight distributions in the handle significantly affect the NMI values of the muscle pairs in the upper limb. For testing the hypotheses, handle mock-ups with five different CMs (centroid, top-rear, top-front, bottom-front, and bottom-rear) were prepared with a 3D printer, and EMG data of the five muscles of the right upper limb were measured for 18 female participants during given vacuuming tasks with the handle mock-ups. Lastly, the measure data were statistically analyzed and the results were discussed with regard to the creation of an ergonomic design guide for the handle of a cordless stick-type vacuum cleaner and further addressing its risks of developing MSDs.

## 2. Materials and Methods

### 2.1. Participants

Eighteen female college students were recruited as participants for testing. Their average age and height were 23.6 years (SD: 1.5) and 164.4 cm (SD: 4.2), respectively. They were all right handers and had used stick-type vacuum cleaners for more than one-year. In addition, no musculoskeletal pain and discomfort on the body was reported on the day of the experiments. All the participants signed an informed consent form and were given a description of the testing procedures. Note that the present study was reviewed and approved by the institutional review board (IRB) of the Dongguk University—Seoul (IRB number: DUIRB-202109-03).

### 2.2. Mock-Up

**Figure 1:**
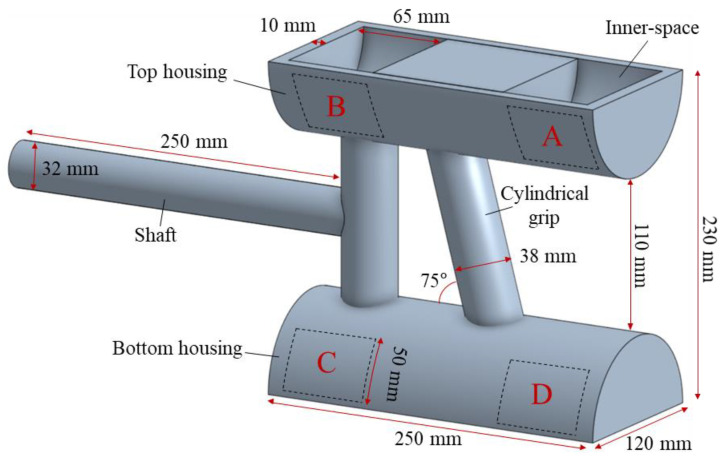
Schematic drawing of the handle mock-up. The letters A, B, C, and D indicate four inner spaces for attaching weights; A: top-rear, B: top-front, C: bottom-front, D: bottom-rear.

A cleaner handle mock-up was manufactured by polylactic acid (PLA) with a 3D printer (Stick, Jeungpyeong-gun, Republic of Korea), as shown in Figure 1. The weight of the mock-up was 800 g and it consisted of three parts: (1) cylindrical grip; (2) shaft; and (3) top and bottom housings. The cylindrical grip was attached between the top and bottom housings and tilted 75° with the housingcis (referring to Dyson V10). In addition, its diameter and length were 38 mm and 114 mm, respectively, which allowed it to fully accommodate females with 95th percentile hand breadth (86 mm) [40] as well as allowing them to produce a maximum power grip force [41]. Next, the shaft was installed in the front part of the mock-up. It was made for helping connect the handle mock-up to a canister (i.e., stick). Lastly, the top and bottom housings were designed to hold the cylindrical grip and shaft. Two inner-spaces (65 mm × 110 mm × 50 mm) were prepared at both ends of each housing (i.e., a total of four inner-spaces in the handle mock-up), as illustrated using dotted lines with the letters A, B, C, and D in Figure 1, and were used for attaching weights.

### 2.3. EMG Measurement

**Figure 2:**
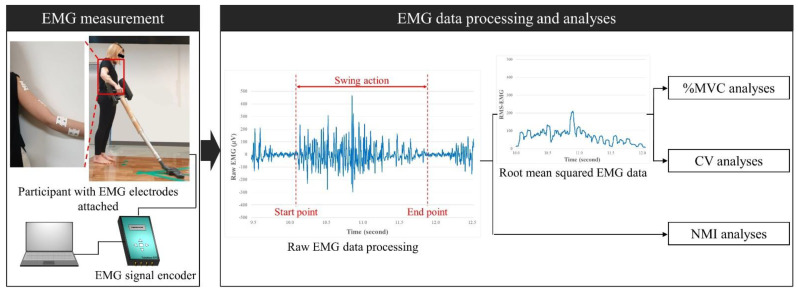
Overview of Electromyography (EMG) measurement and data processing.

EMG was measured from the five muscles of the right upper extremity including the Extensor Carpi Ulnaris (ECU), Brachioradialis (BR), Biceps Brachii (BB), Deltoid Middle (DM), and Upper Trapezius (UT), functioning for wrist extension, forearm flexion, elbow flexion, arm abduction, and shoulder shrug, respectively [6,10,42,43]. EMG data collection and processing were conducted as illustrated in Figure 2. The Telemyo DTS surface EMG system (Noraxon, Scottsdale, AZ, USA) was used as an EMG signal encoder for testing. Disposable EMG electrodes (10 mm diameter with 25 mm inter-electrode spacing; 3M Korea, Seoul, Republic of Korea) were attached on the bellies of the five muscles, as described in Table 1 (recommended by Perotto) [43]—the designated skin areas for attaching the electrodes were prepared according to the surface EMG for noninvasive assessment of muscles (SENIAM) [44]. Note that a reference electrode was attached on the Acromion of the right shoulder. Surface EMG signals were recorded at 1500 Hz (sampling rate), and the noises and artifacts were removed through bandwidth filters ranging from 10 to 500 Hz. Here, separate low and high pass filters were not applied, because it was estimated that there could be relatively less movement artifacts due to the experimental protocol, where vacuuming tasks were conducted in standing posture (only participants’ right upper limbs were in dynamic swing motions). The raw EMG data were processed from the beginning to the end of the onset of a muscle contraction (i.e., one cyclic swing of the arm = forward + backward) (Figure 2). Here, the raw EMG data were managed in two ways. Above all, the raw EMG data were rectified and root mean squared (RMS) with 50 ms moving window (25 ms overlapping) and their means were quantified for normalized EMG analyses. Simultaneously, in addition, the raw EMG data were stored without any transformation for NMI analyses later.

Elbow flexion against a rigid resistance with the shoulder flexed at 90° and the elbow flexed at 90°, in a kneeling position

Maximum voluntary contraction (MVC) was measured to compute %MVC (i.e., normalized EMG). MVCs for the ECU, BR, BB, DM, and UT were recorded in pre-determined static postures differentiated by each muscle (Table 1), according to the measurement protocols of Caldwell et al. [45], Choi and Shin [10], Fedorowich and Cote [46], and Konrad [47]. MVC measurement was repeated twice for each muscle and 5 min rest time was given between the trials. Each measurement lasted five seconds with the status of full exertion in the given static postures, and then the EMG data for three seconds in the middle were taken for quantifying the MVC of each muscle; the data were rectified and root-mean squared for each trial. Lastly, the maximum value of the two measurements were taken as the MVC of the corresponding muscle.

### 2.4. Experimental Design

To investigate the effects of five cordless stick-type vacuum cleaner handles that have different CM locations (i.e., the weight distribution groups: G1, G2, G3, G4, and G5 in Table 2), a total of 13 handles with different weight distributions were implemented for testing. Four weights of 450 g, 150 g, and 2 × 100 g, corresponding to a battery, a motor, a dust cup, and miscellaneous parts, respectively, were employed by referring to the part specifications of three representative cordless stick-type vacuum cleaners available in the market (Dyson V10, LG A958IA, and Samsung VS20R9044SB). They were affixed via Velcro to the top-rear, top-front, bottom-front, and bottom-rear inner-spaces (i.e., A, B, C, and D in Figure 1) of the top and bottom housings of the handle mock-up. The attachment orders were combined to simulate a variety of weight distribution conditions of a cordless stick-type vacuum cleaner handle. All the possible combinations of the four weights (a total of 12 weight distribution conditions) were created, and then the combinations were appropriately grouped (e.g., G2, G3, G4, and G5) according to where their heaviest weight (450 g) was assigned among the A, B, C, and D inner-spaces, as shown in Table 2. Note that one-factor within-subject ANOVA on each weight distribution group (α = 0.05) showed that there was no statistical significance among the weight distribution conditions in each weight distribution group, and thus the combinations were grouped into G2, G3, G4, and G5, as mentioned above. In addition, one extra condition (G1) in which weight was evenly distributed across the handle mock-up (i.e., 200 g: 200 g: 200 g: 200 g) was added to the weight distribution conditions as a reference for testing. The overall weight (excluding a canister and a brush) of the handle mock-up with the weights were maintained at 1.6 kg—this was similar to the weights of the three representative cordless stick-type vacuum cleaners in the market (approximately, 1.7 kg for Dyson V10, 1.6 kg for LG A958IA, and 1.6 kg for Samsung VS20R9044SB).

**Figure 3:**
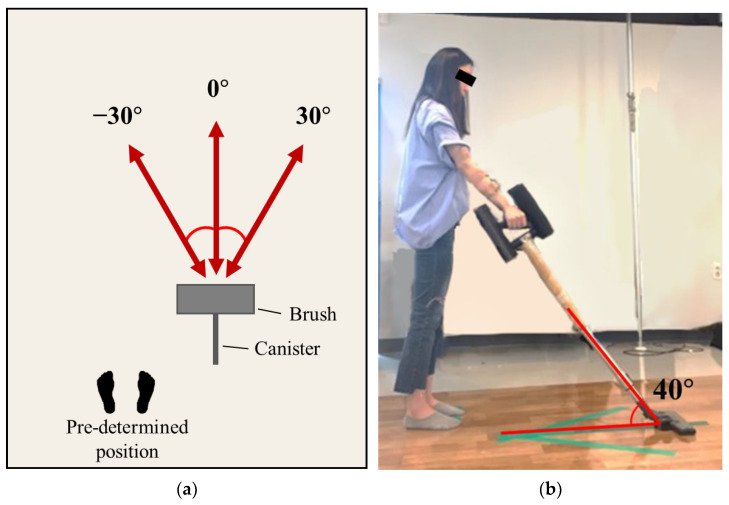
Experimental setting. (**a**) Pre-determined position and arm swing directions for testing; (**b**) Adjustment of canister length.

An experimental space (2.3 m × 1.8 m) was set up and a thin floor mat (coefficient of friction = 0.64) was installed on the floor. The handle mock-up was connected to a canister and a brush (canister + brush = 1.1 kg). Participants were instructed to stand on the pre-determined position in a natural way (Figure 3a) and requested to grasp the handle mock-up using the right hand. They were allowed to adequately adjust the length of the canister between 97 cm to 120 cm so that the canister could maintain a 40° angle with the floor in a standing posture with fully extended elbow (Figure 3b), which helped prevent the canister length from being too long or short for the participants’ stature during the experiments. Cleaning motions were controlled in three directions (−30°, 0°, and 30°), as shown in Figure 3a, and the participants were asked to swing (forward + backward) every two seconds with auditory cueing of a metronome (i.e., 30 arm swings/min).

One-factor within-subject design was used for testing. An independent variable was the weight distribution condition and a dependent variable was the EMG signal. EMG was measured for all the weight distribution conditions (i.e., a total of 13) of the handle mock-up. One experimental trial consisted of 15 swings (3 directions × 5 swings), and two trials were repeated for each weight distribution condition (2 repetitions × 13 weight distribution conditions). The orders of the swing directions and given weight distribution conditions were completely randomized in the experiments, and two-minute rest was given to participants between the trials. Testing was conducted in three steps. First, the experimental information such as objective and procedure was explained to participants. Second, a practice trial was given to each participant so as to help familiarize them with the use of the given handle mock-up. Third, the main study was performed: EMG data for the five designated muscles were recorded while participants conducted the given vacuuming task.

### 2.5. Data Analysis

Since the measured EMG data failed to show statistical significance among the weight distribution conditions within each weight distribution group, the measured EMG data were analyzed in terms of the five weight distribution groups (i.e., G1, G2, G3, G4, and G5 in Table 2) instead. Note that such a large number of levels (i.e., 13 combinations) were intentionally avoided because this could unnecessarily increase the amount of type I errors in ANOVA, which might make the statistical result unreliable. Therefore, the measured EMG data for each weight distribution condition were assigned to the corresponding weight distribution groups: (1) G1 contained 36 measurements (18 participants × 1 weight distribution condition × 2 trials) and (2) G2, G3, G4, and G5 contained 108 measurements, respectively (18 participants × 3 weight distribution conditions × 2 trials). Here, the weight distribution groups were determined according to where their heaviest weight (450 g) was assigned among the A, B, C, and D inner-spaces in the handle mock-up (Table 2), and thus each group indicated the case where CM of the handle mock-up was leaning toward the corresponding inner-space (except G1 as a reference group).

The recorded EMG data were analyzed in two ways. Above all, the RMS EMG data were examined in terms of muscle activity and motor variability. Normalized MVC (i.e., %MVC) values were computed by dividing the RMS EMG data of each muscle by the corresponding MVCs. The %MVC values of the five weight distribution groups were compared with one another in each muscle, and thereby their muscle activities while using the handle mock-ups of different weight distribution groups were investigated from a kinetic point of view. In addition, the coefficient of variations (CV = standard deviation ÷ mean × 100) were computed from the corresponding %MVC values in order to quantify the EMG signal variability of each muscle for the five weight distribution groups. Thus, the normalized variations of muscle performance while using the handle mock-ups with different CMs were observed in terms of motor variability. According to previous studies [19,20,21], high CV value indicates a large range of variability in the EMG signal of a muscle, which means that the muscle has more opportunities for muscular rest (i.e., period of relaxation) during the task execution. On the contrary, a low CV value signifies that there are less opportunities for muscular rest in the EMG signal of a muscle because the muscle was constantly activated during the EMG measurement.

Next, NMI values among the recorded muscles were quantified to determine the extent of functional connectivity between two EMG time series in a muscle pair while using the handle mock-ups of different weight distribution groups. In the present study, the NMI was computed based on the approach detailed in Johansen et al. [34], Kawczynski et al. [36], and Madeleine et al. [39]. The five recorded muscles were paired and thus a total of ten pairs were created as follows: (1) ECU-BR, (2) ECU-BB, (3) ECU-DM, (4) ECU-UT, (5) BR-BB, (6) BR-DM, (7) BR-UT, (8) BB-DM, (9) BB-UT, and (10) DM-UT. The raw EMG data of each muscle were normalized with the corresponding MVCs, and the density functions of the recorded muscles were estimated by constructing the histograms with 40 bins. The NMI value was computed over non-overlapping windows of 500 ms and the mean value was taken to represent the trial. The value of the NMI was determined between 0 and 1, which indicated “no functional connectivity” and “complete connectivity” within the muscle pair, respectively. Here, high NMI value indicates high amounts of EMG signal commonality (i.e., muscle co-contraction) between the muscle pair [30,34,36].

To statistically investigate the effects of the weight distribution groups of the handle mock-up, one-factor within-subject ANOVAs were conducted with α = 0.05 on each of the %MVC, CV, and NMI; the ANOVAs were performed separately for each muscle in the case of the %MVC and CV and for each muscle pair in the case of the NMI. Note that before the ANOVAs, the normality of all the data were confirmed via the Shaprio-Wilks test. The Student-Newman-Keuls (SNK) test was employed as a post-hoc analysis at the same significance level.

## 3. Results

### 3.1. Muscle Activity

**Figure 4:**
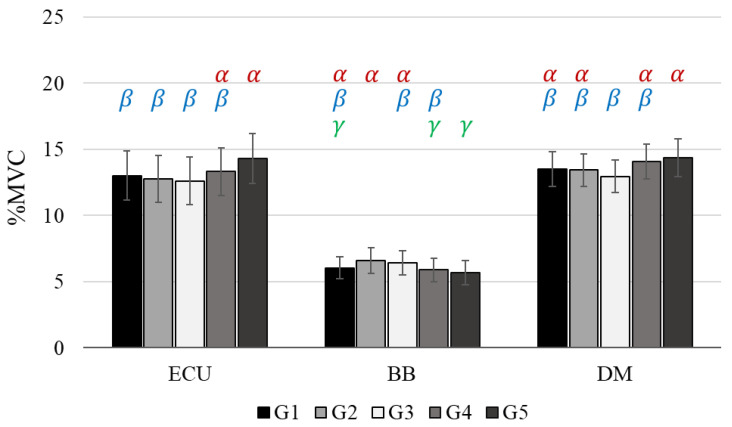
The results of %MVC values for 18 participants. %MVC: %Maximum Voluntary Contraction, ECU: Extensor Carpi Ulnaris, BB: Biceps Brachii, DM: Deltoid Middle. Error bars indicate standard error. The Greek letters indicate statistical significance at 95% confidence level: α > β > γ.

The %MVC values of the ECU, BB, and DM significantly varied as a function of the weight distribution groups (Figure 4, Table 3 and Table 4). Overall, the %MVC values on the ECU and DM increased as the heaviest weight (450 g) moved from the top (i.e., the weight distribution group G2 and G3) to bottom housing (G4 and G5) of the handle mock-up. The SNK test determined that G4 and G5 were categorized into the group with significantly larger %MVC values and G1, G2, G3, and G4 were classified into the group with significantly smaller %MVC values, in both of the muscles. Meanwhile, the reverse trend was found in the %MVC values of the BB. I.e., the %MVC values increased while the heaviest weight moved from the bottom (G4 and G5) to top (G2 and G3) housing of the handle mock-up. The SNK test showed that G1, G2, and G3 were grouped into the highest cluster and G1, G4, and G5 were categorized into the lowest cluster. No significant difference was found between the front and rear areas (i.e., G2 vs. G3 and G4 vs. G5) in the handle, and G1 was classified into both the statistically highest and lowest groups (except the ECU).

### 3.2. Motor Variability

**Figure 5:**
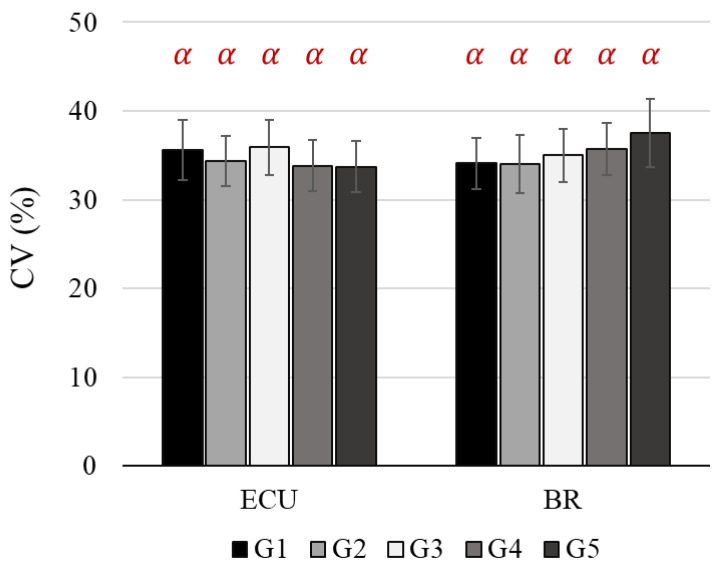
The results of CV values for 18 participants. CV: Coefficient of Variation, ECU: Extensor Carpi Ulnaris, BR: Brachioradialis. Error bars indicate standard error. The Greek letter indicates statistical significance at 95% confidence level.

The CV values on the ECU and BR were altered significantly as the weight distribution groups changed (Figure 5, Table 3 and Table 4). However, the SNK tests failed to classify the weight distribution groups into statistically different clusters on both of the muscles. The CV values showed opposite propensities between the ECU and BR. When the heaviest weight (450 g) moved from the top (G2 and G3) to bottom housing (G4 and G5) of the handle mock-up, the CV values of the ECU decreased but that of the BR increased, as shown in Table 3 and Table 4. On the other hand, the BB, DM, and UT had relatively higher CV values than the ECU and BR in the forearm.

### 3.3. Functional Connectivity

**Figure 6:**
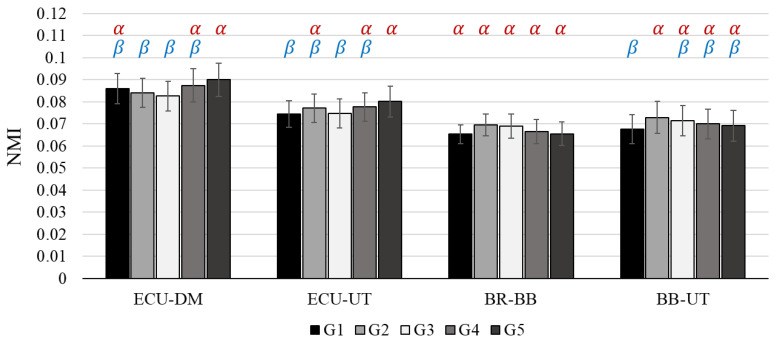
The results of NMI values for 18 participants. NMI: Normalized Mutual Information, ECU: Extensor Carpi Ulnaris, BR: Brachioradialis, BB: Biceps Brachii, DM: Deltoid Middle, UT: Upper Trapezius. Error bars indicate standard error. The Greek letters indicate statistical significance at 95% confidence level: α > β.

The weight distribution groups had significant effects on the NMI values of the ECU-DM, ECU-UT, BR-BB, and BB-UT (muscle pairs), as shown in Figure 6, Table 3 and Table 4. Two different patterns were found in the NMI values of those muscle pairs. First, the NMI values of the ECU-DM and ECU-UT decreased together while the heaviest weight moved from the bottom (G4 and G5) to the top (G2 and G3) housing of the handle mock-up. The SNK tests determined that G4 and G5 were categorized into the cluster with statistically higher NMI values, and G1, G2, G3, and G4 were simultaneously classified into the cluster with statistically lower NMI values on both of the muscle pairs. Second, the NMI values on the BR-BB and BB-UT shared decreasing trends as the heaviest weight moved from the top (G2 and G3) to the bottom (G4 and G5) housing of the handle mock-up. The SNK test for the NMI values of the BB-UT classified G2, G3, G4, and G5 into the highest group and categorized G1, G3, G4, and G5 into the lowest group. However, the SNK test for the NMI values on the BR-BB failed to show statistical significance among the weight distribution groups, despite its similar tendency. Interestingly, meanwhile, G1 was classified into the statistically lowest NMI groups in the muscle pairs that showed statistical significance. In addition, G1 was last in rank across most of the muscle pairs in the present study.

## 4. Discussion

### 4.1. Muscle Activity

The ECU showed significantly higher muscle activities (i.e., %MVC values) when the heaviest weight was attached in the bottom housing of the handle mock-up (i.e., G4 and G5) rather than when it was attached in the top housing (G2 and G3). This can be interpreted as meaning that the ECU needed to exert larger force for using the handle mock-ups of which CMs were around the bottom area. This finding was consistent with the previous study that investigated the effects of weight distributions on a cordless stick-type vacuum cleaner handle in terms of muscle activity [6]. Kang et al. [6] explained the reasons why the %MVC values on the ECU increased as the CM of the handle moved from the top to bottom area, in two ways. First, they addressed that these results could be affected by the fundamental vacuuming motions that occur when using a cordless stick-type cleaner—in particular, with respect to the motion of pulling the handle, in which the bottom part of the handle rotates in the pulling direction around the wrist of the hand that grasps the handle grip and the top part of the handle rotates in the opposite direction of the pulling (i.e., the counterclockwise rotation of the handle around the cylindrical grip in Figure 1). They pointed out that in the case of using the handle with the CM at the bottom (exactly below the hand position grasping the handle grip), the motion of such pulling of the handle may lead to a natural increase of the load on the ECU because the muscle needs to extend the wrist for pulling the handle and directly lift up the most of the handle weight at the same time. Second, they explained this finding with the variation of the distance between the CM of a handle and the wrist (assumed as a pivot) as well. They noted one phenomenon which was likely to occur during vacuuming with a stick-type vacuum cleaner, such that the hand that grasps a cleaner handle may slide up along the cylindrical grip of the handle because the handle body often slides down along the axis of the grip in the hand due to the weight of the handle (i.e., due to gravity). The reason why they were interested in this was because this phenomenon could help elongate the distance between the CM of a handle and the wrist when using the handle with the CM at the bottom and vice versa while using the handle with the CM at the top. Thus, they suggested a mechanism that since the moment arm length between the CM of a cleaner handle and the wrist of the hand (grasping its handle grip) could be a little lengthen while using a handle with the CM at the bottom, the spontaneous torque of the handle for balancing itself from gravity naturally increased during vacuuming [48]. Accordingly, they concluded that more effort of the ECU would be needed to control the elevated torque during vacuuming.

The BB had the reverse patterns with the ECU—i.e., significantly larger %MVC values were observed while using the handle mock-up with the CM at the top (G2 and G3), and significantly smaller %MVC values were found while using the handle mock-up with the CM at the bottom (G4 and G5). In fact, this was expected because these findings were consistent with the laws of physics: the longer the moment arm is, the greater the torque becomes. To be more specific, since users typically hold stick-type cleaner handles at an angle to the floor during vacuuming (like Figure 3b in the present study), the top area of the handle moves further away from the elbow joint and the bottom area of the handle moves closer to the elbow joint—this phenomenon is constantly maintained during vacuuming. Therefore, when the CM of the handle is at the top area (i.e., G2 and G3 in the present study) the moment arm length between the CM and the elbow joint is increased, and when the CM of the handle is at the bottom area (i.e., G4 and G5) the moment arm length between the CM and the elbow joint is shortened. In other words, naturally, relatively larger and smaller torques are required on the BB while pulling (elbow flexion) the handles, respectively. We concluded that this mechanism sufficiently explained the results of the present study, in which the muscle activity of the BB significantly increased when using the handle mock-up with the CM at the top and vice versa when using the handle mock-up with the CM at the bottom during vacuuming.

Like the muscle activities of the ECU, the %MVC values of the DM significantly increased as the CM of the handle mock-up moved from the top (G2 and G3) to the bottom housing (G4 and G5). The reason for this was unclear, but the result was interpretable from two perspectives. First, this phenomenon could be evidence that the DM assists in pulling a cordless stick-type vacuum cleaner handle during vacuuming. In general, it is observed that the lateral deviation (i.e., abduction) of the shoulder is naturally required for the movements of the upper limb during vacuuming with a stick-type vacuum cleaner. This constantly occurs, especially while pulling its handle, and becomes more apparent when a change of directions exists in arm swing. Here, we can estimate that this phenomenon is not a simple kinematic movement as a part of the swing motion but a necessary motion to pull the cleaner handle during vacuuming. The reason is that it is informed that the DM is one of the representative agonist muscles working for shoulder abduction [43], and there have been clues that the shoulder abduction may contribute to the pulling mechanism of the arm [49,50]. For example, the DM could help pull the handle with abducting the shoulder, as the ECU extended the wrist and simultaneously lift up the handle mock-up. In this context, this mechanism can explain why the EMG amplitude of the DM increased when using the handle mock-up with the CM at the bottom in the present study. Second, the motions of the upper arm and shoulder during vacuuming are likely to become relatively smaller when using a cordless stick-type cleaner handle with the CM at the top. This is because the muscle activity (i.e., EMG amplitude) of the DM used to be increased when the extent of the shoulder abduction becomes larger [51,52]. For example, the lateral movement of the shoulder could be decreased (i.e., motion efficiency) while using a handle with the CM at the top rather than while using a handle with the CM at the bottom. However, this interpretation needs to be cautiously made because the kinematic motion of the shoulder was not considered in the scope of the present study. Thus, further study is warranted to validate this.

### 4.2. Motor Variability

Among the recorded five muscles, only the ECU and BR in the forearm showed significantly different motor variabilities as a function of the weight distribution groups. Above all, the CV values of the ECU tended to be declined as the CM of the handle mock-up moved from the top (G2 and G3) to the bottom housing (G4 and G5). From the perspective of traditional motor variability studies, this finding can be translated as meaning that the ECU had less opportunities for muscular rest while using the handle mock-up with the CM at the bottom [19,20]—i.e., the ECU could be exposed to a relatively higher risk of occurrence of MSDs especially in the prolonged use of a cordless stick-type cleaner handle with the CM at the bottom, because it is well-known that repetitive and constant muscle use is one of the major causes of developing MSDs [8,9]. We deduced that the cause for this was strongly tied up with the reasons why the %MVC values of the ECU increased when using the handle mock-up with the CM at the bottom. This is because such small CV values of the ECU could imply that the ECU needs to exert itself more frequently and relatively longer when using a handle of which the CM is at the bottom, such as in this study, so as to (1) lift up the elevated weight of the handle bottom part and (2) control the increased spontaneous torque of the handle mock-up. On the contrary, the CV values of the BR tended to be elevated as the CM of the handle mock-up moved from the top (G2 and G3) to the bottom housing (G4 and G5). This indicated that the BR had less opportunities for muscular rest while using the handle mock-up with the CM at the top. We found the reason for this phenomenon to be the fact that the BR is one of the agonist muscles that flex the elbow like the BB [43]—as the BB did, that is to say, the %MVC values of the BR could be affected significantly by the moment arm length between the CM of the handle mock-up and the elbow joint, while pulling the handle mock-up. As mentioned earlier, the muscle activity of the BB, while using the handle mock-up with the CM at the top housing (G2 and G3), the moment arm length between the CM and the elbow joint can be increased. Given the circumstances, such small CV values of the BR can be interpreted as meaning that there would be a potential risk that the BR may have been exerted more frequently and relatively longer to overcome the extended moment arm length when pulling the handle mock-up with the CM at the top, which could significantly decrease the opportunities for muscular rest of the BR. In sum, although these findings could be regarded as evidence that the muscle activities of the ECU and BR in the forearm were more sensitively affected by the CM locations of the handle mock-up, as compared to the BB, DM, and UT, these were unlikely to be noticeably strong because the CV values of both the muscles failed to show significant differences among the weight distribution groups in the SNK tests (a post-hoc analysis).

Aside from the above findings, two notable points were additionally found in the motor variability analysis. First of all, overall the motor variabilities (CV range: approximately, 33~51%) of the recorded five muscles in the present study were relatively small, in comparison with the motor variabilities of other tasks with muscle activities: (1) approximately 46~65% on the shoulder and neck muscles (the anterior deltoid, lower trapezius, middle trapezius, and upper trapezius) of young females (average age: 24.1 years) during computer tasks (e.g., reading, typing) with a laptop and a dual monitor desk-top workstation in 9 min [30] and (2) approximately 40~70% on the upper limb muscles (the extensor carpi radialis, anterior deltoid, lower trapezius, middle trapezius, and upper trapezius) of young females (age range: between 18 and 30 years) during keyboard typing with cycling [31]. This can be interpreted in two ways from a biomechanical point of view. First, vacuuming with a cordless stick-type vacuum cleaner requires relatively more constant activation of the related muscles. Note that, simultaneously, this may indicate that the vacuuming motion itself using a canister is fundamentally difficult. Second, a user could be exposed to a relatively higher risk of developing MSDs in the long-term use of a cordless stick-type vacuum cleaner. However, this interpretation needs to be cautiously made because simple comparisons of numbers were done without detailed investigations, and there have not been any absolute threshold values of motor variability to assess whether a given CV value of the muscle activity is low or not [46,53]. Next, the ECU and BR in the forearm had relatively smaller CV values (approximately, 10% gap on average) than the BB, DM, and UT, in the present study. This finding can be translated as meaning that the ECU and BR were more constantly activated than the BB, DM, and UT during vacuuming. I.e., literally, the ECU and BR could be more exposed to a relatively higher risk of occurrence of MSDs than the BB, DM, and UT in the prolonged use of such a type of cleaner. Although it is known that a stick-type vacuum cleaner is typically used for short periods of time as well as by housekeepers primarily at home [6,42], this would still be considered a potential risk of the ECU and BR in the long-term use of a cordless stick-type vacuum cleaner.

### 4.3. Functional Connectivity

The NMI values of the muscle pairs ranged from 0.06 to 0.10. I.e., overall, the likelihoods that two muscles (in every muscle pair) were functionally connected (co-contraction) during vacuuming were between 6 to 10% in the present study. These NMI values were estimated to be a rather large when considering that the vacuuming is regarded as a dynamic task. In general, the NMI values of static tasks are relatively larger than that of dynamic tasks because low muscle connectivity is a beneficial muscle strategy during active work [30,38]. To be specific, the NMI values of the present study tended to be larger than the NMI values of both the static and dynamic tasks introduced in previous studies: (1) 0.01~0.05 on the shoulder and neck muscle pairs (the anterior deltoid, lower trapezius, middle trapezius, and upper trapezius) during computer tasking (e.g., reading, typing) with a laptop and a dual monitor desk-top workstation [30]; (2) 0.10~0.20 on the muscle pairs in the forearm (the extensor carpi ulnaris, extensor carpi radialis, flexor carpi ulnaris, and flexor digitorum superficialis) during dynamic tracking tasks using compensatory and pursuit display [38]; (3) 0.01~0.04 on the upper limb muscle pairs (biceps brachii, middle deltoid, supraspinatus, lower trapezius, middle trapezius, and upper trapezius) during a repetitive pointing task at shoulder height [28]; and (4) 0.00~0.03 on the muscle pairs on the dominant side of the upper limb (the flexor digitorum superficialis, extensor digitorum, extensor carpi radialis, lower trapezius, upper trapezius, and cervical erector spinae) during texting on a smartphone and computer typing [39]. This phenomenon can have two meanings from a biomechanical point of view. First, the co-contractions between muscles could occur relatively more prevalently during vacuuming with a cordless stick-type vacuum cleaner [28,34]. Simultaneously, however, this may mean that the motion itself required for vacuuming with a canister is stuck to such a phenomenon. Second, the vacuuming with a cordless stick-type cleaner is likely to not only cause relatively more muscle fatigue but also to be exposed to a relatively higher risk of developing MSDs in its prolonged use [34,36,37]. Note that, however, given that the limited number of the NMI studies on muscle activities have been conducted, further reviews via more comparison studies are warranted rather than drawing hasty conclusions based on the above interpretations.

The NMI values of the ECU-DM and ECU-UT muscle pairs were increased as the CM of the handle mock-up moved from the top (G2 and G3) to the bottom housing (G4 and G5). This indicated that the muscles in those pairs were more functionally connected while using the handle mock-up with the CM at the bottom. The reason for each muscle pair was examined: first of all, we deduced that the result for the ECU-DM was strongly associated with the muscle activities of the ECU and DM. This was because the %MVC values of both the ECU and DM were simultaneously increased while using the handle mock-up with the CM at the bottom in the present study. For example, this means that both of the muscles were highly activated together nearly at the same time and thus the co-contractions between the ECU and DM could have occurred more prevalently. Yoon et al. [31] supported this mechanism but explained that this phenomenon could actually come up in the opposite order; in other words, more functional connectivity between the ECU and DM could occur first and then the fatigue symptoms are spread in the muscles, which naturally increases the EMG amplitudes (%MVC values) of the ECU and DM. Meanwhile, the reason for the above NMI variations of the ECU-UT was unclear, because there was no evidence that the UT was highly activated together with the ECU while using the handle mock-up with the CM at the bottom, as compared with the ECU-DM. However, given that the %MVC values of the UT were maintained at approximately 10% regardless of the weight distribution groups of the handle mock-up, we estimated that it would be likely that such NMI values of the ECU-UT were determined by the influence alone of the %MVC variations on the ECU; i.e., the NMI values of this muscle pair may have been elevated because the EMG amplitude of the ECU increased when using the handle mock-up of which CM was at the bottom. This can be supported by the fact of the present study that the %MVC values of the ECU and the NMI values of the ECU-UT shared similar rankings among the weight distribution groups of the handle mock-up excluding the ranking of G1.

On the contrary, the NMI values of the BB-UT muscle pair were declined while the CM of the handle mock-up moved from the top (G2 and G3) to the bottom housing (G4 and G5). The muscles in this muscle pair were more functionally connected when using the handle mock-up with the CM at the top. We estimated that the cause for such NMI variations of the BB-UT was similar to the cause for the variations in the NMI values of the ECU-UT. This was because the %MVC values of the BB and the NMI values of the BB-UT shared similar rankings among the weight distribution groups of the handle mock-up excluding the ranking of G1, as shown in the relationship between the ECU and ECU-UT. That is, it would be likely that the NMI values of the BB-UT were strongly affected by the variations in the %MVC values of the BB that showed statistical significance as a function of the weight distribution groups of the handle mock-up. Given the circumstances, this allows the following interpretation for the present study: since the BB needed a greater force to pull the handle mock-up with the CM at the top, the BB and UT were more functionally connected to assist the exertion of the BB.

On the other hand, the NMI values of the BR-BB also varied significantly depending on the weight distribution groups of the handle mock-up. There seemed to exist a decreasing trend in the NMI values of the BR-BB when the CM of the handle mock-up moved from the top (G2 and G3) to the bottom housing (G4 and G5), though the SNK test failed to show statistical significance as a function of the weight distribution groups. In fact, we judged that the above NMI variations of the BR-BB were natural. This was because (1) both of the muscles were the agonist muscles of elbow flexion (i.e., elbow flexors) as well as (2) they shared similar rankings from one another among the weight distribution groups of the handle mock-up, in the %MVC and CV values of the present study. Song et al. [54] confirmed that the muscle activities of the BR and BB are not only more sensitive to the changes of the weight or CM of an object in the hand but also increased together when the elbow flexion begins. In other words, the co-contractions between the BR and BB could occur more prevalently when pulling the handle mock-up with the CM at the top, because their muscle activities are sensitively affected by the extended moment arm length between the CM of the handle mock-up and the elbow joint at the same time.

Lastly, relatively low NMI values were observed with the handle mock-up of G1 (i.e., the weight distribution group in which weight was evenly distributed across the handle mock-up). Across all of the significant muscle pairs, G1 was not only categorized into the statistically lowest group, but also was always last in rank except for the ECU-DM. Furthermore, G1 had relatively lower NMI values even in the muscle pairs that failed to show statistical significance. This can be interpreted as meaning that overall a relatively small amount of co-contraction between the muscles in the muscle pairs occurs when using the handle mock-up of G1 in the present study. The reason for this was unclear. However, we can deduce the plausible causes for this finding. First, this could be because of the fact that the closer the CM of a handle is to the hand for grasping the handle, the less the rotation of the handle due to gravity occurs [48]. As mentioned earlier, when the CM of a handle moves away from the hand that grasps the handle, the spontaneous torque of the handle for balancing itself from gravity could be naturally increased. Accordingly, this allows for less muscle force to be required to control such torque when using a handle of which weight is evenly distributed across the handle (the CM is at centroid). Second, given that the above phenomenon was prominent, especially in the ECU-UT and BB-UT, we can estimate the reason based on the role of the UT and its EMG amplitude. Researchers reported that the UT is regarded as a stabilizer muscle that typically helps improve the joint stability of the shoulder and neck and prevent injuries to them [55,56,57]. Simultaneously, they emphasized that the smaller the EMG amplitude value of the UT is, the more stable movement the shoulder and neck joints have. In this context, this allows the following explanation for the present study: if the %MVC values of the UT were relatively low when using the handle mock-up of G1, a relatively small number of co-contraction between the UT and other muscles (i.e., the ECU and BB) may occur because the shoulder and neck more stably moved when using the handle mock-up of G1. This was consistent with the result of the present study that the %MVC of the UT was last in rank when using the handle mock-up of G1, although it failed to show statistical significance (it is marginal; *p* = 0.0931). This was also supported by Kang et al. [6], who studied the effect of the weight distribution of a cordless stick-type cleaner handle and thereby reported that the significantly lowest EMG amplitude of the UT was found while using a handle of which weight was evenly distributed across the handle.

### 4.4. Implications and Limitations

The implications of the present study can be summarized as follows. First, there seemed to exist trade-offs between the %MVC values of the muscles that showed statistical significance (i.e., the ECU, BB, and DM) as the weight distribution groups changed. This was because there were inverse relationships between the %MVC values for those muscles (the ECU and DM vs. the BB). When fixing G1 in the middle and comparing the %MVC values of G2 and G3 with that of G4 and G5 side by side, these trends became apparent. In short, this allows that the evaluation for the effects of the weight distribution groups may be at odds, in terms of EMG amplitude. Second, the weight distribution groups of the handle mock-up would not have noticeable strong effects on the CV values of the EMG signals for the recorded muscles. This was supported by three clues: (1) only the CV values of two muscles, the ECU and BR, showed statistical significance as a function of the weight distribution groups; (2) the SNK tests (post-hoc analysis) for the CV values of those two muscles even failed to show statistical significance among the CM locations of the handle mock-up; and (3) their *F*-values were not that large to be considered sufficiently significant effect sizes. We estimated that this would be evidence that the motor variabilities of the recorded muscles were more affected by the inherent difficulty of the vacuuming motion itself with a stick-type vacuum cleaner rather than the CM locations of the handle mock-up. Third, there were inverse relationships between the NMI values of the muscle pairs that showed statistical significance as a function of the weight distribution groups, like the %MVC analysis. Given the circumstances, we judged that the patterns of the NMI values were strongly associated with that of the %MVC values. This was because relatively high NMI values of the muscle pairs were found primarily in the CM locations of the handle mock-up where the muscles belonging to the muscle pairs were highly activated in the EMG signals (i.e., elevated %MVC values) in the present study. In fact, this was expected because such elevated NMI values of the muscle pair could indicate that fatigue was spread in the muscles in the pair and thereby their EMG amplitudes were increased [31]. Lastly, there would be a potential risk of developing MSDs in the prolonged use of a cordless stick-type vacuum cleaner. The reason for this was because relatively low CV and high NMI values were found during the vacuuming in the present study. Although these resulted from simple numerical comparisons with the other studies as well, as there have been no absolute threshold values of motor variability and functional connectivity for evaluating the extent of corresponding biomechanical risks, and this still means that a user could be exposed to a relatively higher risk of occurrence of MSDs in the long-term use of a cordless stick-type vacuum cleaner. To sum up, studies for design strategies and scientific countermeasures are still warranted in order to mitigate and systematically manage such potential risks of developing MSDs when using a cordless stick-type vacuum cleaner.

All things considered, G1 is comprehensively recommended as the optimum CM location for the handle of a cordless stick-type vacuum cleaner. Three reasons support this recommendation. First, less muscle effort from the muscles in the forearm (especially, the ECU) would be needed in controlling a handle of G1 during vacuuming. As mentioned earlier, this was because the closer the CM of a handle is to the hand that grasps the handle, the smaller the spontaneous torque of the handle for balancing itself from gravity may become. That is, this indicates that the load of the muscles in the forearm could be consequently reduced because less muscle effort to control the spontaneous torque is needed when using the handle of G1. Note that, given the mechanism, we expect that similar effects are likely to occur in the antagonist muscles (e.g., the Triceps Brachii and Deltoid Anterior) for pulling a handle (i.e., agonist muscles that contribute to pushing a handle) although they were out of the scope of the present study. Second, there was no evidence that G1 was more negative than the other weight distribution groups of the handle mock-up from the CV analysis of the present study. This was determined based on the aforementioned finding that the weight distribution groups would not have statistically noticeable strong effects on the CV values. I.e., the weight distribution groups had either nearly no impacts or almost similar impacts from one another on the motor variability results of the present study. In this context, we concluded that G1 had a similar impact to the other weight distributions of the handle mock-up, in terms of motor variability. Lastly, the results of the present study addressed that G1 was likely to be the best CM location for the handle of a cordless stick-type vacuum cleaner, in terms of functional connectivity. G1 was not only last in rank across most of the muscle pairs in the present study, but was also categorized into the statistically lowest group of the NMI values on the muscle pairs that showed statistical significance depending on the CM locations of the handle mock-up. This could mean that while using the handle of G1, less functional connectivity between the related muscles occurs, meaning that a user may be exposed to a relatively lower risk of occurrence of MSDs in the long-term use of the handle. In other words, G1 could have a design benefit in the prolonged use of a cordless stick-type vacuum cleaner from a biomechanical point of view. Note that the applications of the current findings and design recommendations should be conducted within the research scope of the present study; the applications beyond the scope of this study should be cautiously done.

Further studies are needed to address the knowledge gap and generalization of the present study. First, more advanced experimental set-up, which can include more actual vacuuming conditions and situations in real life, is necessary to improve the practicality and fidelity of this study. Since the present study was still in its early stages, the experimental design had the following limitations: (1) the effect of the vacuuming suction power was dismissed; (2) the vacuuming task consisted of only simplified motions (pushing and pulling in three directions); and (3) only short-term testing was conducted. Thus, given the circumstances, considering various real vacuuming situations such as implementing suction power and including more vacuuming actions would be useful in complementing the lack of realism and validating the findings of the present study. Second, more muscles relevant to vacuuming motions using a cordless stick-type vacuum cleaner need to be used for testing. The present study employed only five muscles, most of which were the agonist muscles that are used for pulling the handle mock-up. Unfortunately, this unbalanced selection of target muscles may lead to missing key research findings as well as causing limited or biased interpretation on the results, which could interfere with the generalization of the study. Therefore, expanded studies that consider the balanced selection of agonist and antagonist muscles are warranted to improve the reliability and significance of this study. Third, grip strength measurement would provide useful information to advance the interpretation of the findings. In the present study, we regarded the variation of hand grip strength for holding a cleaner handle as a phenomenon that could naturally happen during vacuuming with a stick-type vacuum cleaner, and thus the grip strength was not considered a parameter that had to be observed separately with interest during testing. However, it has been demonstrated that grip strength for holding a handle is associated with the muscle activities (especially, the ECU and BR) of the upper limbs [58,59]. Thus, further studies to find out the extent to which the grip strength contributes to the muscle activities of the upper limb could be useful for advancing the validity of the present study. Lastly, studies including different gender and age groups are needed. The present study recruited only female college students in their 20s as participants, and thus the impact of the findings may be limited within their gender and age groups. Naturally, more comprehensive studies involving different gender and age groups are necessary, because such studies would contribute to better generalizability of the findings for the present study.

## 5. Conclusions

This study examined the muscle activities, motor variability, and functional connectivity for the muscles of the upper limbs as a function of CM locations in a handle of a cordless stick-type vacuum cleaner. The findings of the present study are as follows. First, the results of the %MVC values showed that the effects of the weight distribution groups of the handle mock-ups may be at odds in terms of muscle activity. There seemed to exist inverse relationships between the trends of the %MVC values for the ECU, BB, and DM, although the %MVC values of those muscles were significantly varied as a function of the weight distribution groups. Second, the signals’ CV values of the recorded muscles may not be a critical metric for evaluating the impact of the weight distribution groups of the handle mock-up. The CV values of the ECU and BR were significantly altered depending on the CM locations of the handle mock-up, but failed to show statistical significance in the post-hoc analyses; i.e., the weight distribution groups would not have statistically noticeable effects on the CV values. Third, there would be a potential risk of developing MSDs in the prolonged use of a cordless stick-type vacuum cleaner. Although this was concluded from simple numerical comparisons with the other studies as mentioned above, relatively lower CV and higher NMI values were found during the vacuuming in the present study, meaning that a user could be exposed to relatively higher risks of occurrence of MSDs. All things considered, G1 would be recommended as the optimal CM location for the handle of a cordless stick-type vacuum cleaner. This was supported by the fact that G1 was beneficial in terms of muscle activity and that the NMI values of G1 were last in rank across most of the muscle pairs in the present study.

## Figures and Tables

**Table 1:** Electrode locations and MVC measurement protocols for the recorded muscles.

Muscle	Electrode Location	MVC Protocol
Extensor Carpi Ulnaris	Just above the shaft of ulna	Wrist extension against dorsal resistance of hand with the upper arm straight down, the elbow flexed at 90°, the forearm pronated, and neutral wrist, in a seated position
Brachioradialis	Midway between biceps tendon and lateral epicondyle along flexor crease	Forearm flexion against a rigid resistance with the upper arm straight down and the elbow flexed at 90°, in a seated position
Biceps Brachii	The bulk of the muscle in mid-arm	Elbow flexion against a rigid resistance with the shoulder flexed at 90° and the elbow flexed at 90°, in a kneeling position
Deltoid Middle	Halfway between the tip of the acromion and the deltoid tubercle	Shoulder abduction against a rigid resistance with the shoulder abducted at 90° in a seated position
Upper Trapezius	At angle of neck and shoulder	Shoulder elevation against rigid resistance

MVC: Maximum Voluntary Contraction.

**Table 2:** Weight distribution groups and conditions of a handle mock-up.

Group Number	Condition Number	Weight Assigned to Each Inner-Space (Unit: g)			
		Inner-Space A	Inner-Space B	Inner-Space C	Inner-Space D
G1	1	200	200	200	200
G2	2	450	150	100	100
	3	450	100	150	100
	4	450	100	100	150
G3	5	150	450	100	100
	6	100	450	150	100
	7	100	450	100	150
G4	8	150	100	450	100
	9	100	150	450	100
	10	100	100	450	150
G5	11	150	100	100	450
	12	100	150	100	450
	13	100	100	150	450

**Table 3:** The %MVC, CV, and NMI values as a function of weight distribution groups.

Dependent Variable		Mean (Standard Error)				
		Weight Distribution Group				
		G1	G2	G3	G4	G5
%MVC (unit: %)	ECU	13.0 (1.9)	12.8 (1.8)	12.6 (1.8)	13.3 (1.8)	14.3 (1.9)
	BR	10.6 (1.1)	11.5 (1.2)	11.2 (1.2)	10.6 (1.2)	10.6 (1.3)
	BB	6.0 (0.8)	6.6 (1.0)	6.4 (0.9)	5.9 (0.9)	5.7 (0.9)
	DM	13.5 (1.3)	13.4 (1.2)	13.0 (1.2)	14.1 (1.3)	14.4 (1.4)
	UT	9.7 (1.9)	10.6 (2.1)	10.2 (2.0)	10.4 (1.9)	11.0 (2.1)
CV (unit: %)	ECU	35.6 (3.3)	34.3 (2.8)	35.9 (3.1)	33.8 (2.9)	33.7 (2.9)
	BR	34.1 (2.9)	34.0 (3.2)	35.0 (3.0)	35.7 (3.0)	37.5 (3.8)
	BB	44.9 (3.4)	45.4 (3.4)	45.6 (3.3)	46.4 (3.3)	47.6 (3.7)
	DM	44.3 (3.0)	43.5 (3.3)	44.3 (3.5)	44.4 (3.4)	43.3 (3.2)
	UT	49.6 (3.9)	50.6 (4.3)	49.0 (4.1)	49.7 (4.4)	47.8 (4.7)
NMI	ECU-BR	0.089 (0.008)	0.092 (0.008)	0.090 (0.008)	0.087 (0.008)	0.091 (0.009)
	ECU-BB	0.062 (0.005)	0.064 (0.006)	0.062 (0.006)	0.063 (0.006)	0.064 (0.007)
	ECU-DM	0.086 (0.007)	0.084 (0.007)	0.083 (0.007)	0.087 (0.007)	0.090 (0.008)
	ECU-UT	0.074 (0.006)	0.077 (0.007)	0.075 (0.006)	0.078 (0.006)	0.080 (0.007)
	BR-BB	0.065 (0.004)	0.069 (0.005)	0.069 (0.005)	0.067 (0.005)	0.065 (0.005)
	BR-DM	0.082 (0.006)	0.083 (0.006)	0.082 (0.006)	0.083 (0.006)	0.083 (0.006)
	BR-UT	0.072 (0.006)	0.077 (0.006)	0.075 (0.006)	0.074 (0.006)	0.075 (0.007)
	BB-DM	0.076 (0.005)	0.077 (0.005)	0.076 (0.006)	0.078 (0.006)	0.077 (0.006)
	BB-UT	0.068 (0.007)	0.073 (0.007)	0.071 (0.007)	0.070 (0.007)	0.069 (0.007)
	DM-UT	0.090 (0.008)	0.093 (0.008)	0.090 (0.008)	0.094 (0.008)	0.094 (0.009)

%MVC: %Maximum Voluntary Contraction, CV: Coefficient of Variation, NMI: Normalized Mutual Information, ECU: Extensor Carpi Ulnaris, BR: Brachioradialis, BB: Biceps Brachii, DM: Deltoid Middle, UT: Upper Trapezius.

**Table 4:** Ranking of weight distribution groups.

Dependent Variable		Ranking of Weight Distribution Groups	*F*(4, 68)	*p*-Value
%MVC (unit: %)	ECU	G5 ^α^, G4 ^α,^^β^ > G4 ^α,^^β^, G1 ^β^, G2 ^β^, G3 ^β^	4.27	**0.0038**
	BR	G2, G3, G1, G5, G4	2.40	0.0583
	BB	G2 ^α^, G3 ^α,^^β^, G1 ^α,^^β,γ^ > G3 ^α,^^β^, G1 ^α,^^β,γ^, G4 ^β,γ^ > G1 ^α,^^β,γ^, G4 ^β,γ^, G5 ^γ^	6.81	**0.0001**
	DM	G5 ^α^, G4 ^α,^^β^, G1 ^α,^^β^, G2 ^α,^^β^ > G4 ^α,^^β^, G1 ^α,^^β^, G2 ^α,^^β^, G3 ^β^	3.65	**0.0094**
	UT	G5, G2, G4, G3, G1	2.08	0.0931
CV (unit: %)	ECU	G3 ^α^, G1 ^α^, G2 ^α^, G4 ^α^, G5 ^α^	2.78	**0.0335**
	BR	G5 ^α^, G4 ^α^, G3 ^α^, G1 ^α^, G2 ^α^	2.67	**0.0394**
	BB	G5, G4, G3, G2, G1	0.84	0.5017
	DM	G4, G1, G3, G2, G5	0.43	0.7892
	UT	G2, G4, G1, G3, G5	0.90	0.4680
NMI	ECU-BR	G2, G5, G3, G1, G4	1.10	0.3653
	ECU-BB	G5, G2, G4, G3, G1	1.06	0.3829
	ECU-DM	G5 ^α^, G4 ^α,^^β^, G1 ^α,^^β^ > G4 ^α,^^β^, G1 ^α,^^β^, G2 ^β^, G3 ^β^	4.89	**0.0016**
	ECU-UT	G5 ^α^, G4 ^α,^^β^, G2 ^α,^^β^ > G4 ^α,^^β^, G2 ^α,^^β^, G3 ^β^, G1 ^β^	3.23	**0.0174**
	BR-BB	G2 ^α^, G3 ^α^, G4 ^α^, G5 ^α^, G1 ^α^	2.74	**0.0356**
	BR-DM	G2, G4, G5, G1, G3	0.41	0.7989
	BR-UT	G2, G3, G5, G4, G1	1.72	0.1562
	BB-DM	G4, G2, G5, G3, G1	0.74	0.5654
	BB-UT	G2 ^α^, G3 ^α,^^β^, G4 ^α,^^β^, G5 ^α,^^β^ > G3 ^α,^^β^, G4 ^α,^^β^, G5 ^α,^^β^, G1 ^β^	3.59	**0.0102**
	DM-UT	G5, G4, G2, G1, G3	1.91	0.1184

%MVC: %Maximum Voluntary Contraction, CV: Coefficient of Variation, NMI: Normalized Mutual Information, ECU: Extensor Carpi Ulnaris, BR: Brachioradialis, BB: Biceps Brachii, DM: Deltoid Middle, UT: Upper Trapezius. The Greek letters indicate statistical significance at 95% confidence level (α > β > γ). *F*(4, 68) is the result of ANOVA *F*-test and *p*-value also refers to ANOVA. The bold texts in the *p*-values indicate statistical significance at 95% confidence level.

## Data Availability

The data presented in this study are available on request from the corresponding author.
